# Population pharmacokinetics and target attainment of pretomanid in rifampicin-resistant tuberculosis patients

**DOI:** 10.1038/s41598-026-46217-2

**Published:** 2026-03-31

**Authors:** Bern-Thomas Nyang’wa, Ilaria Motta, Ronelle Moodliar, Varvara Solodovnikova, Shakira Rajaram, Mohammed Rasool, Catherine Berry, Zhonghui Huang, Geraint Davies, David A.J. Moore, Frank Kloprogge

**Affiliations:** 1 Médecins sans Frontières, Public Health Department, Amsterdam, The Netherlands; 2 Institute for Global Health, University College London, London, UK; 3 University College London, MRC-CTU, London, UK; 4 THINK (TB and HIV Investigative Network, Durban, South Africa; 5 Republican Scientific and Practical Centre of Pulmonology and Tuberculosis, Minsk, Belarus; 6 Clinical HIV Research Unit (CHRU), Department of Internal Medicine, School of Clinical medicine, Faculty of Health Sciences, Wits Health Consortium (WHC), University of Witwatersrand, Johannesburg, South Africa; 7 Manson Unit, Public Health Department, Médecins sans Frontières, London, UK; 8 UCL-GOSH Institute of Child Health, University College London, London, UK; 9 Department of Clinical Infection, Microbiology and Immunology, University of Liverpool, Liverpool, UK; 10 London School of Hygiene and Tropical Medicine, Clinical Research Department, London, UK

**Keywords:** Population pharmacokinetics, Pretomanid, Rifampicin resistant tuberculosis, Probability target attainment, Diseases, Drug discovery, Medical research, Microbiology

## Abstract

Pretomanid is a key component of the bedaquiline, pretomanid, linezolid with or without moxifloxacin (BPaL/M) regimen recommended for treatment of rifampicin-resistant tuberculosis (RR-TB). To support dose optimization and efficacy interpretation, we developed a pretomanid population pharmacokinetic (PK) model and evaluated exposure and probability of target attainment (PTA). Ninety-four RR-TB patients received daily oral pretomanid at 200 mg, and plasma samples were collected at multiple time points. Pretomanid concentrations were quantified using high-performance liquid chromatography-tandem mass spectrometry and PK modeling was performed using nlmixr2 in R. A one-compartment model with first-order absorption and elimination, and fat free mass allometric scaling best described the data. Typical clearance was 3.10 L/h (32.9%CV), median AUC₀₋₂₄ was 64,000 (31,000– 140,000) ng·h/ml, and the median maximum inter-dose concentration (Cmax) was 3,000 (1,000– 6,000) ng/ml. Pretomanid MICs for *Mycobacterium tuberculosis* in the TB-PRACTECAL trial were consistently below the provisional critical concentration, with a median of 0.125 mg/L. PK-Pharmacodynamic (PD) simulations indicated that nearly all participants achieved drug exposures exceeding %fT > MIC target, consistent with the regimen’s clinical efficacy across the study population. However, the AUC/MIC target was not achieved. We developed a pretomanid population PK model and facilitated exploring robustness of PK-PD targets for PTA. Our study confirmed the clinical relevance of the time dependent index and target, but further investigation is needed to determine whether the 167 AUC/MIC target is valid in patients or using translational pre-clinical experiments, especially within the context of combination therapy.

**Trial registry**: Clinical Trials.gov, TRN: NCT04081077, Registration date: 4 September 2019.

**Supplementary Information:**

The online version contains supplementary material available at 10.1038/s41598-026-46217-2.

## Introduction

Tuberculosis (TB) is the leading cause of death from a single infectious agent, claiming approximately 1.2 million lives globally each year. The emergence of drug-resistant strains poses a significant challenge to TB control efforts^1^. The World Health Organization recommends a six-month, fully oral regimens comprising bedaquiline, pretomanid and linezolid, with or without moxifloxacin (BPaL/M) as the preferred treatment approach for rifampicin-resistant tuberculosis (RR-TB)^2^. These regimens and together with bedaquiline, pretomanid, linezolid, and clofazimine (BPaLC), were investigated for the treatment of RR-TB in the TB-PRACTECAL randomised controlled clinical trial^3,4^.

Pretomanid is a nitroimidazooxazine antimycobacterial approved for the treatment of TB as part of a BPaL regimen. It is extensively metabolized through reductive and oxidative metabolism but no major pathway has been identified thus far. Around 20% is metabolised through cytochrome P450-3A and 1% appears in urine as unchanged pretomanid. At an oral dose of 200 mg, steady state pharmacokinetic (PK) parameters in fasted individuals are as follows: Cmax 1.7 mg/L, Tmax of 4.5 h, T1/2 16 h. A high-fat, high-calorie meal increases Cmax by 76% and AUC by 88% when compared to the fasting state^5–8^, therefore in a fed, steady state the Cmax would increase to around 3.0 mg/L.

Pretomanid inhibits mycolic acid biosynthesis, thus disrupting cell wall production in actively replicating *Mycobacterium tuberculosis*. It also kills non-replicating bacteria in anaerobic environments by generating reactive nitrogen species including nitric oxide^9^. Maximum efficacy is achieved with a daily dose of 200 mg, increased toxicity but not increased efficacy is experienced with higher doses in early bactericidal studies^10^; doses of 50 mg, 100 mg and 150 mg captured incremental efficacy^11^. At a dose interval of less than 48 h, pretomanid efficacy PK-Pharmacodynamic (PD) can be defined by area under the free drug concentration curve (fAUC/MIC) or cumulative percentage of the dosing interval that the free drug concentration exceeds the MIC (fT > MIC) parameters^12^. However, pretomanid protein binding in vivo has not yet been determined, approaches have included using total drug concentration or 85% binding^7,12,13^.

This study aimed to investigate pretomanid PK characteristics amongst rifampicin resistant tuberculosis patients receiving combination therapy. Patients were enrolled in the PKPD sub-study of the TB-PRACTECAL trial and PK exposure measures and prospectively collected *Mycobacterium tuberculosis* susceptibility data were assessed against previously reported PK-PD indices and targets.

## Results

All of the patients enrolled in the PKPD sub-study that contributed pretomanid plasma concentration-time data were retained for analyses. 36% of the 94 study participants were female and the median age was 36 years (range: 19–71 years) (Table 1). The total number of timed pretomanid plasma samples included in the population PK analysis was 952, 86 samples were collected before the first dose and 866 samples after the first dose. A total of 234 samples were below the limit of quantification (BLQ), of which 151 samples were collected after treatment completion, rendering 9.5% of the pretomanid concentration BLQ during treatment. All partial data was retained for analyses and samples below limit of quantification during treatment were treated as missing data and the remaining values treated as if they came from a full distribution as in the so-called M1 method, since the percentage of post-dose below limit of quantification samples during treatment was less than 10%. Observed pretomanid plasma concentrations ranged between 19.1 and 11,566 ng/ml, the median trough concentration was 1,789 ng/ml, with an interquartile range of 1126–2689 ng/ml.

**Table 1:** Baseline characteristics of the study participants.

	Pretomanid PK	TB-PRACTECAL
Sample size (*n*)	94	552
Age (y)	36 (19–71)	35 (15–72)
Baseline weight (kg)	56.8 (39.2–144.4)	56.9 (29.7–144.4)
Body mass index (kg/m^2)	19.7 (14.3–47.1)	19.7 (12.5–47.2)
Fat free mass (kg)	45.5 (28.6–75.5)	46 (22.9–77.1)
BUN (mmol/L)	3.6 (1.7–8.5)	3.4 (1.1–9)
ALT (IU/L)	19.5 (4–113)	19 (4–168)
AST (IU/L)	22 (4–82)	24 (4–108)
Total protein (g/L)	77 (61–118)	79 (56–118)
Creatinine clearance (mL/min)	105.4 (43.4–243.8)	108.4 (23.2–342.9)
Female, n (%))	34 (36.2)	223 (40.4)
Caucasian race, n (%)	40 (42.6)	112 (20.3)
Black race, n (%)	52 (55.3)	201 (36.4)
Asian race, n (%)	1 (1.1)	238 (43.1)
Other race, n (%)	1 (1.1)	1 (0.2)
HIV positive, n (%)	39 (41.5)	153 (27.7)
BPaLM, n (%)	38 (40.4)	151 (27.4)
BPaLC, n (%)	30 (31.9)	126 (22.8)
BPaL, n (%)	26 (27.7)	123 (22.3)
SoC, n (%)	–	152 (27.5)

Median (min-max) if not stated otherwise.

*BUN* blood urea nitrogen, *ALT* alanine transaminase, *AST* aspartate aminotransferase.

A one-compartment first order absorption and elimination model best described the observed pretomanid PK time series data. Random effects on clearance and central volume of distribution explained inter-individual variability and a combined residual error model was used to characterise the unexplained variability (Table 1 and appendix 1).

Allometric body size scaling on clearance and volume of distribution, was embedded a priori. However, the choice of using fat free mass (FFM) was made as it improved the base model fit better than weight or BMI. Step-wise covariate modelling rendered female gender, black race, and ‘BPaL regimen’ on volume of distribution in forward first step analysis (*p* < 0.05, per degree of freedom). However, none of these were significant in the backward elimination step (*p* < 0.001, per degree of freedom) (Appendix 2).

**Fig. 1:**
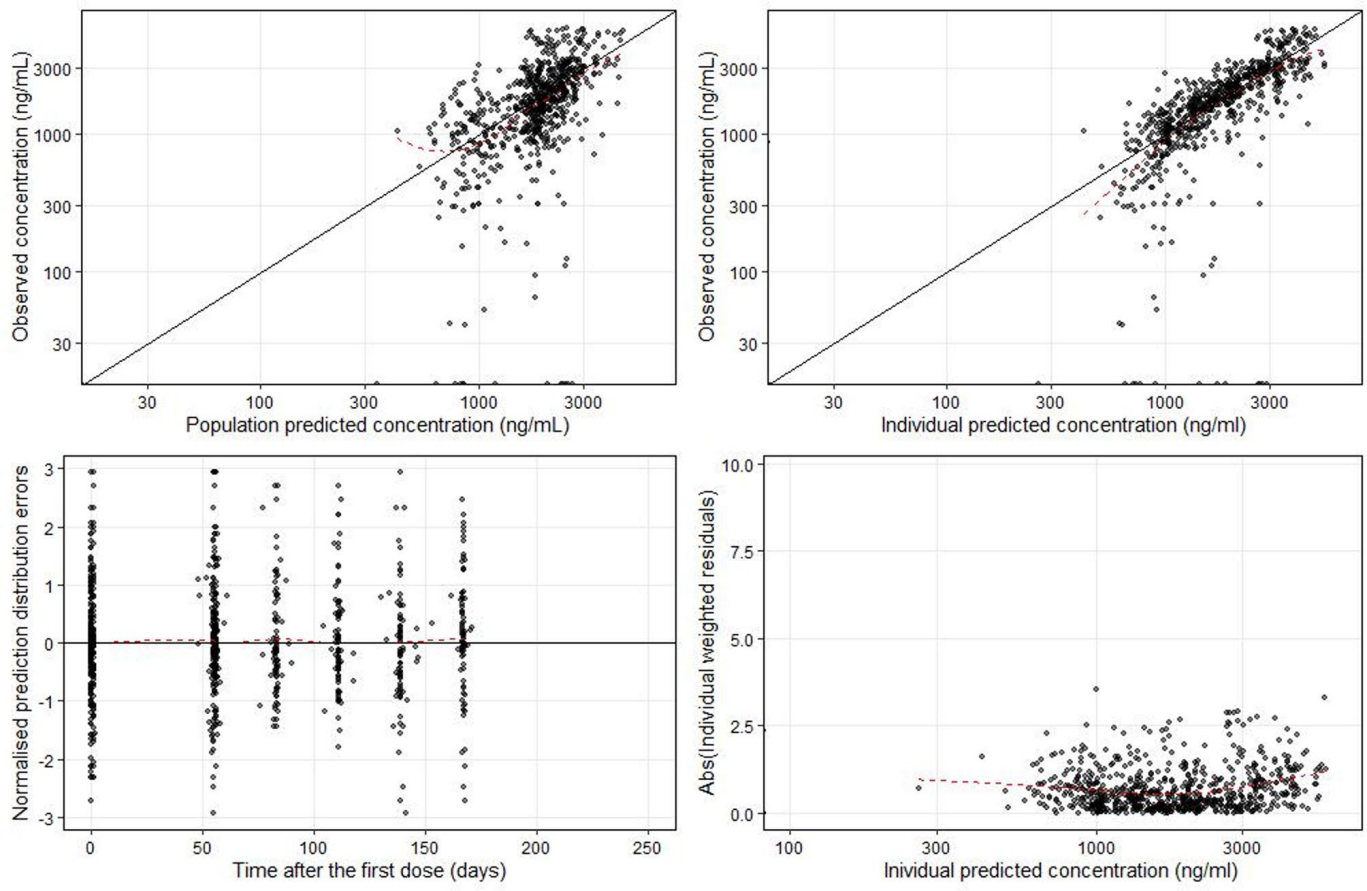
Pretomanid population pharmacokinetic model goodness of fit plots. Dots represent observations, solid black lines represent identity lines and the red dashed lines represent locally estimated scatterplot smoothing.

**Fig. 2:**
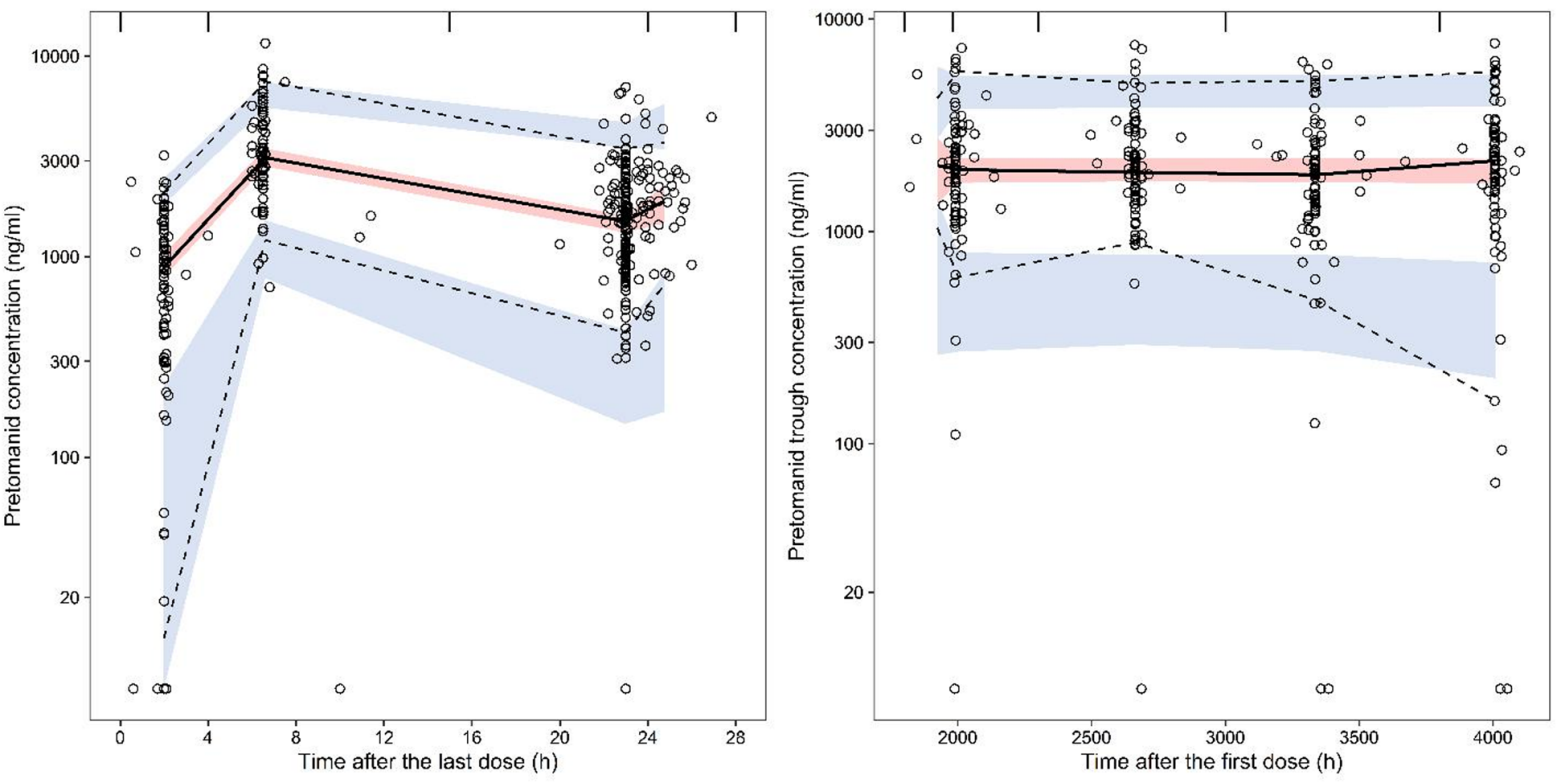
Visual predictive check, presented as time after last dose of the day 1 and week 8 visit (left panel) and time after first dose for the subsequent visits (right panel). Open circles represent the observed data, with dashed and solid lines presenting the 95% percentiles and median of the observed data. The blue and red shaded areas represent the 90% confidence intervals of model predicted 95% percentiles and median of the simulated data.

Goodness-of-fit plots for the pretomanid population PK model showed no significant bias from the unity line, indicating that the model predicted individual and population values closely matched the observed PK data, except for some observed values that fell far below the predictions (Fig. 1). Model validation using a visual predictive check (VPC) and individual model fit, confirmed the predictive accuracy of the pretomanid model (Fig. 2 and Appendix 3). Pretomanid population PK model and secondary parameters are shown in Tables 2 and 3.

**Table 2:** Estimated pretomanid population pharmacokinetic parameters.

Parameter	Parameter estimate (%RSE)	Bootstrap 95% CI
	Final model	
Fixed-effect parameters		
k_a_ (hr^− 1^)	0.316 (19.6)	0.203–0.492
CL/F (L/hr)	3.10 (3.35)	2.87–3.33
V/F (L)	102 (2.45)	81.4–127
Random-effect parameters		
CL/F %CV, [shrinkage %]	32.9 [9.65]	–
V/F %CV, [shrinkage %]	33.6 [36.7]	–
Residual error parameters		
Proportional	0.322	
Additive (mg/L)	0.368	

*k*_*a*_ absorption rate constant, *CL/F* elimination clearance, *V*_*c*_*/F* central volume of distribution, *Q/F* inter-compartmental clearance, *V*_*p*_*/F* peripheral volume of distribution, *%RSE* percentage relative standard error.

**Table 3:** Secondary pharmacokinetic parameters derived with the pretomanid model.

	Median	Range
AUC _0−24_ (µg×h/L)	64,000	30,853–138,179
C_trough_ (µg/L)	2000	65–7702
C_max_ (µg/L)	3,000	1382–6351

*Cmax* maximum pretomanid concentration, *ctrough* concentration just before the next dose was administered, *AUC* area under the plasma concentration-time curve.

**Fig. 3:**
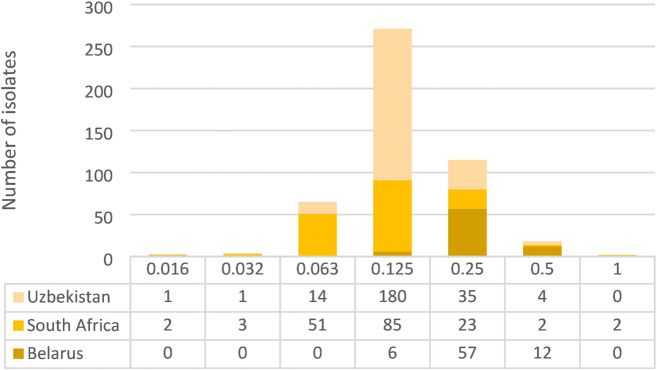
Distribution of *Mtb* baseline isolates across various pretomanid MICs (mg/L) in the TB-PRACTECAL trial.

The distribution of *Mycobacterium tuberculosis* MICs for pretomanid tested using MGIT from 478 TB-PRACTECAL study participants was disaggregated by country of enrolment (Fig. 3). The median MIC was 0.125 mg/L and the interquartile range from 0.125 to 0.25 mg/L. 100% of the isolates were below the provisionally set critical concentration of 1 mg/L^14^ or 2 mg/L^15^. Only two isolates, both from South African sites had a baseline MIC of 1.0 mg/L.

**Fig. 4:**
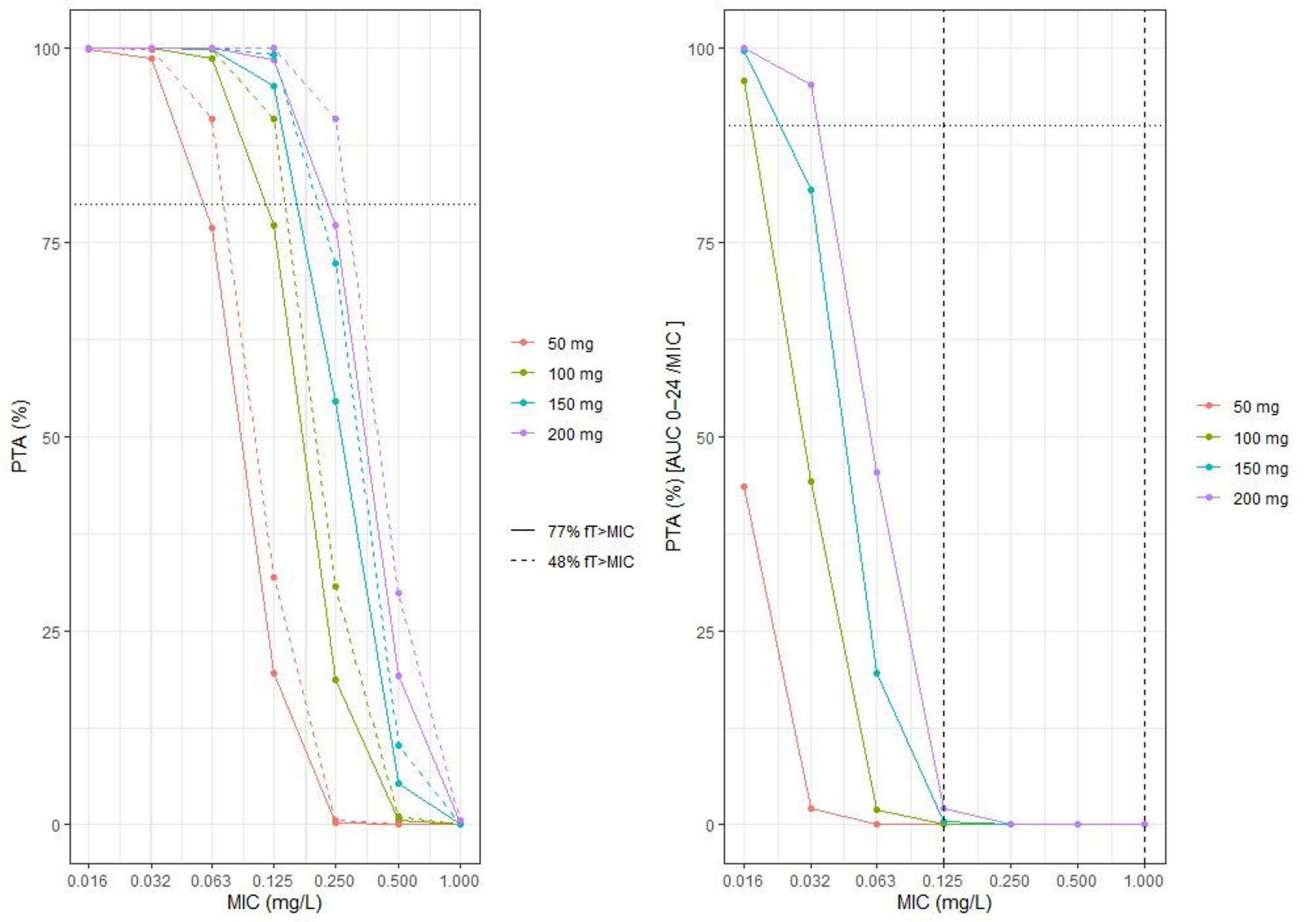
PTA plot for day 14 fT%> MIC (left) and fAUC_0−24_/MIC (right). Both assumed 85% plasma protein binding and a PK-PD target of 167 was selected for fAUC_0−24_/MIC.

Assuming 85% plasma protein binding, a 200 mg dose resulted in 80% or higher PTA fAUC0-24/MIC of 167 at an MIC of 0.032 mg/L and below, (Fig. 4) with lower target achievement in higher protein binding assumptions (Appendix 4). PTA stochastic simulations (*n* = 2,000) using fT > MIC showed that both target of 77% and 48%, associated with 1.59 and 1 log10 kill respectively, were met at 200 mg dosing at MICs of 0.125 and 0.250 mg/L (Fig. 4). Only at the lowest MIC of 0.032 mg/L would a 200 mg dose attain both fAUC0-24/MIC and fT > MIC targets.

At the pretomanid median MIC at baseline in the TB-PRACTECAL trial of 0.125 mg/L, 99.67% of patients would have had drug exposures above %fT > MIC target of 77%, while only 50% would have reached the fAUC/MIC target of 167.

## Discussion

A population pharmacokinetic model for pretomanid was developed using longitudinal data spanning the entire duration of their six-month treatment from the largest single-study cohort to date, which included participants from both South Africa and Belarus. Previous individual studies either recruited patients from a single country or investigated pharmacokinetic time series at a specified time window during their treatment^16^. These results also confirm the adequate pretomanid exposure in rifampicin resistant TB regimens of BPaLM and BPaL which are the currently recommended regimens by WHO^2^.

A one-compartment first order absorption and elimination model with allometric scaling of FFM on both clearance and volume of distribution best described pretomanid PK characteristics. The clearance in our study was estimated at 3.10 L/hr, this is similar to clearances which have been previously published^7,16–19^. The only covariate in our study was FFM (Table 2), concurring with the allometry body size scaling that has been previously reported in other studies using weight on volume and FFM on clearance^7^ or weight on both volume and clearance^16^.

Food administration, particularly high calorie, high fat meal, has been demonstrated to significantly increase pretomanid exposure^8,16^. Our study participants were encouraged to eat before taking medicine. There was no standardised meal, observation, or recording of the type of meal consumed, and therefore current exposures are expected to be representative of real-world scenarios.

Co-administration of potent CYP450 inducers such as efavirenz, lopinavir/ritonavir and rifamycins reduce pretomanid exposure^6,16^. However, these drug classes were contraindicated in the TB-PRACTECAL trial. All the 39 participants living with HIV (42% of total study participants) were on integrase inhibitors and nucleotide reverse transcriptase inhibitor antiretroviral regimens; since HIV was not a significant covariate no further exploration of individual drugs’ effect on the PK model was done. Both moxifloxacin and clofazimine are metabolised in the liver^20,21^. However, including BPaLM and BPaLC as covariates on clearance did not improve the model fit significantly, suggesting none or limited impact of the accompanying anti-TB drugs in the regimen on pretomanid exposure.

In our study 72% of the participants had an MIC of equal to or lower than 0.125 mg/L. Whilst the MIC in this study was measured using MGIT, pretomanid MIC on solid phase media Middlebrook 7H11 has been reported within a similar range at 0.03125 to 0.25 mg/L^22^. At the standard 200 mg daily dose that was used in the TB-PRACTECAL study, assuming 85% protein binding, all isolates within with MIC of 0.250 mg/L or lower, making up 95% of the participants in TB-PRACTECAL, would have achieved the maximal efficacy %fT > MIC targets for a 1-log10 kill (Fig. 4). When using fAUC/MIC as PK-PD target at 167 and the trial’s dose of 200 mg daily, adequate exposure would only be achieved for strains with an MIC of 0.032 mg/L or below (Fig. 4).

One of the limitations of the study was the lack of closely observing food intake. However, since the exposure results are within previously published ranges, it may suggest that the food intake was adequate. The trial excluded patients with moderate liver and renal function abnormality, which makes it less representative of the full spectrum of RR-TB patients.

Pretomanid when given at 200 mg daily in combination with bedaquiline, linezolid with/without moxifloxacin or clofazimine results in adequate exposure. This is the case also in HIV coinfected patients taking antiretroviral treatment consisting of integrase inhibitors and nucleoside reverse transcriptase inhibitors. Assumed protein binding and type of PK-PD index alter the interpretation of probability of target attainment significantly. Our study confirmed the clinical relevance of the time dependent index and target of 77% f%T/MIC, however the 167 *f*AUC/MIC target was not achieved.

## Materials and methods

Pretomanid concentration time series data was available from a nested pharmacokinetic-pharmacodynamic study in TB-PRACTECAL, a randomized controlled trial with rifampicin resistant tuberculosis patients^3,4,23^. Written informed consent was obtained from all participants before recruitment into the TB-PRACTECAL trial and again before any data or sample collection as part of the PRACTECAL-PKPD sub-study. PRACTECAL-PKPD’s first objective was to develop drug exposure metrics for bedaquiline, pretomanid, linezolid, moxifloxacin and clofazimine. The efficacy objectives were to establish an exposure-response model for each drug and regimen to both bactericidal activity and long-term treatment outcomes. The safety objective was to investigate the exposure-toxicity relationship of each drug. All three investigational study arms contained pretomanid as part of the BPaL backbone which was administered alone or with moxifloxacin or clofazimine. Bedaquiline was administered daily for 2 weeks at 400 mg, then at 200 mg three times a week for 22 weeks, linezolid was administered daily at 600 mg for 16 weeks, then at 300 mg daily for the remaining 8 weeks, whilst pretomanid was administered at 200 mg daily for 24 weeks. Clofazimine in the BPaLC arm was administered at 100 mg daily for 24 weeks and moxifloxacin was administered at 400 mg daily for 24 weeks in BPaLM arm.

Blood samples for pretomanid plasma concentration quantification were collected on Day 1 (0, 2 and 23 h), Weeks 8 (predose, 6.5 and 23 h), 12, 16, 20, 24, 32 and 72 post randomisation visits. Veinous blood was collected from the participants’ ante-cubital fossa, centrifuged at 1000 G for 5 min within 30 min of blood drawing. The supernatant plasma was aspirated and pipetted into two equal aliquots approximately 1.5 mL each and stored in temperature of max − 20 °C within 60 min of collection, it was then transferred to dry ice if transport was required and stored in a − 80 °C freezer. At defined intervals, these frozen samples were shipped on dry ice to a GCLP laboratory for quantification. Pretomanid concentrations were quantified using a high-performance liquid chromatography-tandem mass spectrometry. The lower limit of quantification for pretomanid was 7ng/mL.

All data manipulations and analyses were done in R v4.1.2 using nlmixr2, an open-source packages for nonlinear mixed-effect modelling. A list of all R-pakages is listed in appendix 5. Pretomanid concentration time series data were fitted using a first-order conditional estimation with interaction (FOCE-I) algorithm, whilst Inter-individual variability (IIV) at parameter level described variability between patients and residual variability (RV) at observation level described random variability.

Most pretomanid population PK models in the public domain use one-compartment to describe their data^7,16–19^. Consequently, one and two-compartment linear models were evaluated using combined, proportional, additive and log-transformed residual error models. Estimated and fixed first order and transit absorption models were tested to describe pretomanid absorption and random effects on elimination clearance and distribution volume were evaluated.

A matrix comprising covariates and individual eta estimates for clearance and volume of distribution from the base model was used to assess both correlation and collinearity among variables. Allometric body size scaling was applied a-priori to clearance and volume parameters, with fixed exponents of 0.75 for clearance and 1 for volume^24^. Baseline bodyweight, body mass index and fat free mass were compared and the variable realising the largest drop in objective function value compared to a model without body size scaling was selected^25^. Covariate selection was performed using a stepwise approach: forward inclusion was based on a significance threshold of *p* < 0.05 (ΔOFV > 3.84 per degree of freedom), followed by backward elimination using a stricter criterion of *p* < 0.001 (ΔOFV > 10.83 per degree of freedom) to retain only those covariates that significantly enhanced model performance.

Goodness-of-fit plots were used to assess how well the model predicted individual and population values closely matched the observed PK data. Model validation was also performed using visual predictive check (VPC) plots. The shrinkage, relative standard error, and variability value including omega and sigma value were also used to assess the precision and robustness of the model.

Minimum inhibitory concentrations were determined using a routine testing concentration set (1, 0.5, 0.25, 0.125, 0.063, 0.032 mg/L) in MGIT. Testing was performed using a higher (8, 4, 2 mg/L) or lower (0.016 mg/L) testing concentration set if required. The results from all participants from the TB-PRACTECAL trial were summarised by country of enrolment and the median and interquartile range reported.

PTA of pretomanid PK-PD thresholds at doses of 50 mg, 100 mg, 150 mg and the TB-PRACTECAL dose of 200 mg daily were evaluated using 2,000 Monte Carlo simulations and patient level characteristics from the observed participants in this study, accounted for protein binding at 85%-95%^7,12,13^. The fAUC/MIC target was set at 167, which is associated with > 2 log10 reduction in CFU counts. The fT%> MIC targets required for bacteriostatic, 1-log10 kill and 1.59- log10 kill (80% maximum effect), at 22%, 48%, and 77%, were also investigated^12^.

All clinical activities were conducted in accordance with International Council for Harmonisation’s (ICH) Good Clinical Practice and laboratory investigations were conducted in accordance with Good Clinical and laboratory Practice.

## Supplementary Information

Below is the link to the electronic supplementary material.

Supplementary Material 1

## Data Availability

Deidentified data will be available to researchers upon a written request to the Medical Director, Médecins sans Frontières, Operational Centre Amsterdam, the Netherlands.
