# Supplementary material for: Population pharmacokinetics and target attainment of pretomanid in rifampicin-resistant tuberculosis patients
Source: Sci Rep. 2026 Mar 31;16:15255. doi: 10.1038/s41598-026-46217-2 (PMC13180996; doi:10.1038/s41598-026-46217-2)
Supplement: Supplementary file 1 — Supplementary Material 1 [file 41598_2026_46217_MOESM1_ESM.pdf]

Title: Population pharmacokinetics and target attainment of pretomanid in rifampicin-resistant Tuberculosis patients

Running title: Pretomanid PK and PTA in RR-TB patients

*Bern-Thomas Nyang'wa [1,2], Ilaria Motta[3], Ronelle Moodliar[4], Varvara Solodovnikova[5], Shakira Rajaram[6], Mohammed Rasool [6], Catherine Berry[7], Zhonghui Huang[8], Geraint Davies[9], David Moore[10], *Frank Kloprogge[2]

[1] Médecins sans Frontières, Public Health Department, Amsterdam, The Netherlands

[2] Institute for Global Health, University College London, London, United Kingdom

[3] University College London, MRC-CTU, London, United Kingdom

[4] THINK (TB & HIV Investigative Network) Durban, South Africa.

[5] Republican Scientific and Practical Centre of Pulmonology and Tuberculosis, Minsk, Belarus.

[6] Clinical HIV Research Unit (CHRU), Wits Health Consortium (WHC), Department of Internal Medicine, School of Clinical medicine, Faculty of Health Sciences, University of Witwatersrand, Johannesburg, South Africa.

[7] Médecins sans Frontières, Manson Unit, Public Health Department, London, United Kingdom

[8] UCL-GOSH Institute of Child Health, University College London, London, United Kingdom

[9] University of Liverpool, Department of Clinical Infection, Microbiology and Immunology, Liverpool, United Kingdom

[10] London School of Hygiene and Tropical Medicine, Clinical Research Department, London, United Kingdom

*** Corresponding authors**

Bern-Thomas Nyang'wa; bern.nyangwa@london.msf.org

ORCID: 0000-0001-9915-5878

Frank Kloprogge: f.kloprogge@ucl.ac.uk

ORCID: 0000-0001-7213-4559

Supplementary appendix

Appendix 1: R code for the pretomanid model

```
# Pretomanid population pharmacokinetic model code (nlmixr2)
ini({
        lka <- -1.15259732127294
label("Absorption rate")
lcl <- 1.13008124732802
label("Clearance")
lvc <- 4.62170931536226
label("Central volume of distribution")
prop.err <- c(0, 0.321731821549103)
add.err <- c(0, 367.831309683669)
covffmPow1 <- fix(0.75)
covffmPow2 <- fix(1)
eta.cl ~ 0.102674627530168
eta.vc ~ 0.10705836808838
 })

model({
ka <- exp(lka)
cl <- exp(lcl + eta.cl + logFFM * covffmPow1)
vc <- exp(lvc + eta.vc + logFFM * covffmPow2)
linCmt() ~ prop(prop.err) + add(add.err)
 })
```

Appendix 2: Continuous covariate and parameter correlation matrix.

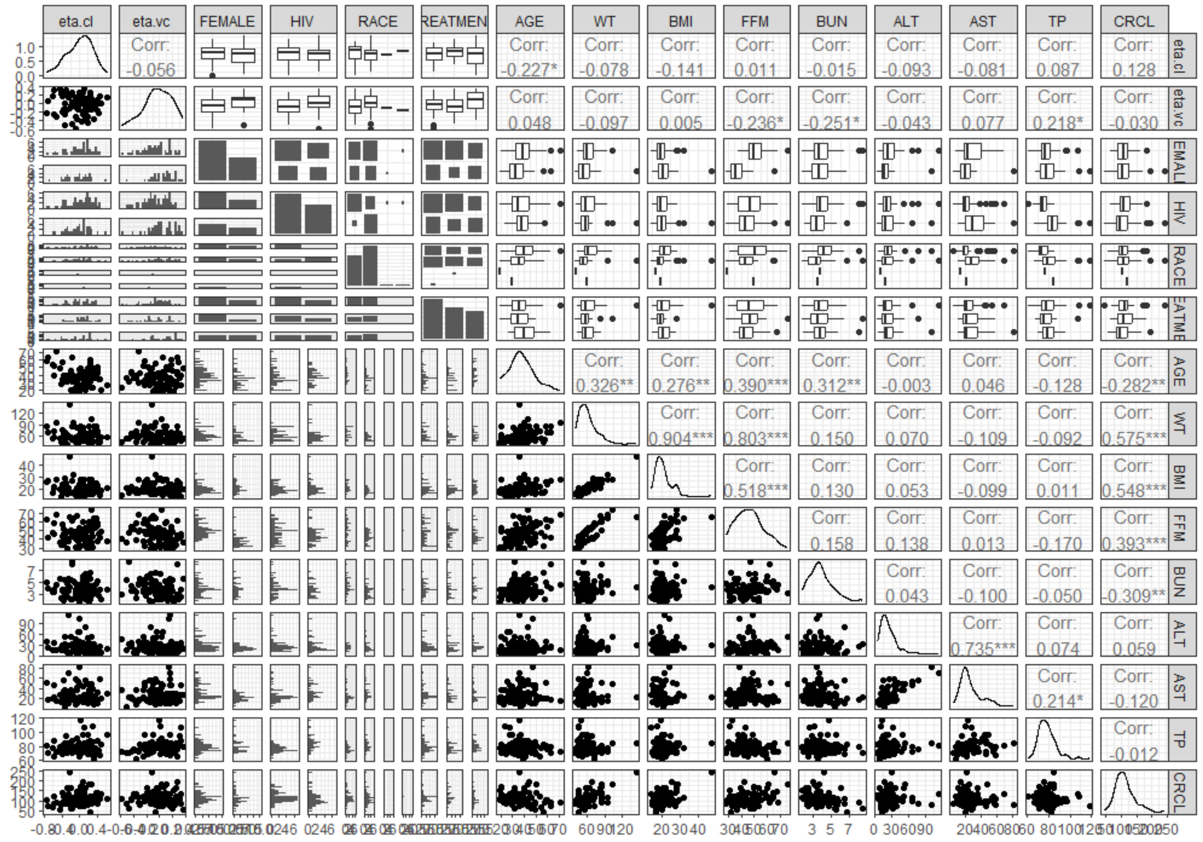

Covariate vs parameter matrix on base model. WT: weight, BMI: body mass index, FFM: fat free mass, BUN: blood urea nitrogen, ALT: alanine transaminase, and AST: aspartate aminotransferase, TP: total protein, CRCL: estimated creatinine clearance, random.g: BPaLM, BPaLC or BPaL arm.

Appendix 3: Individual model fit plots.

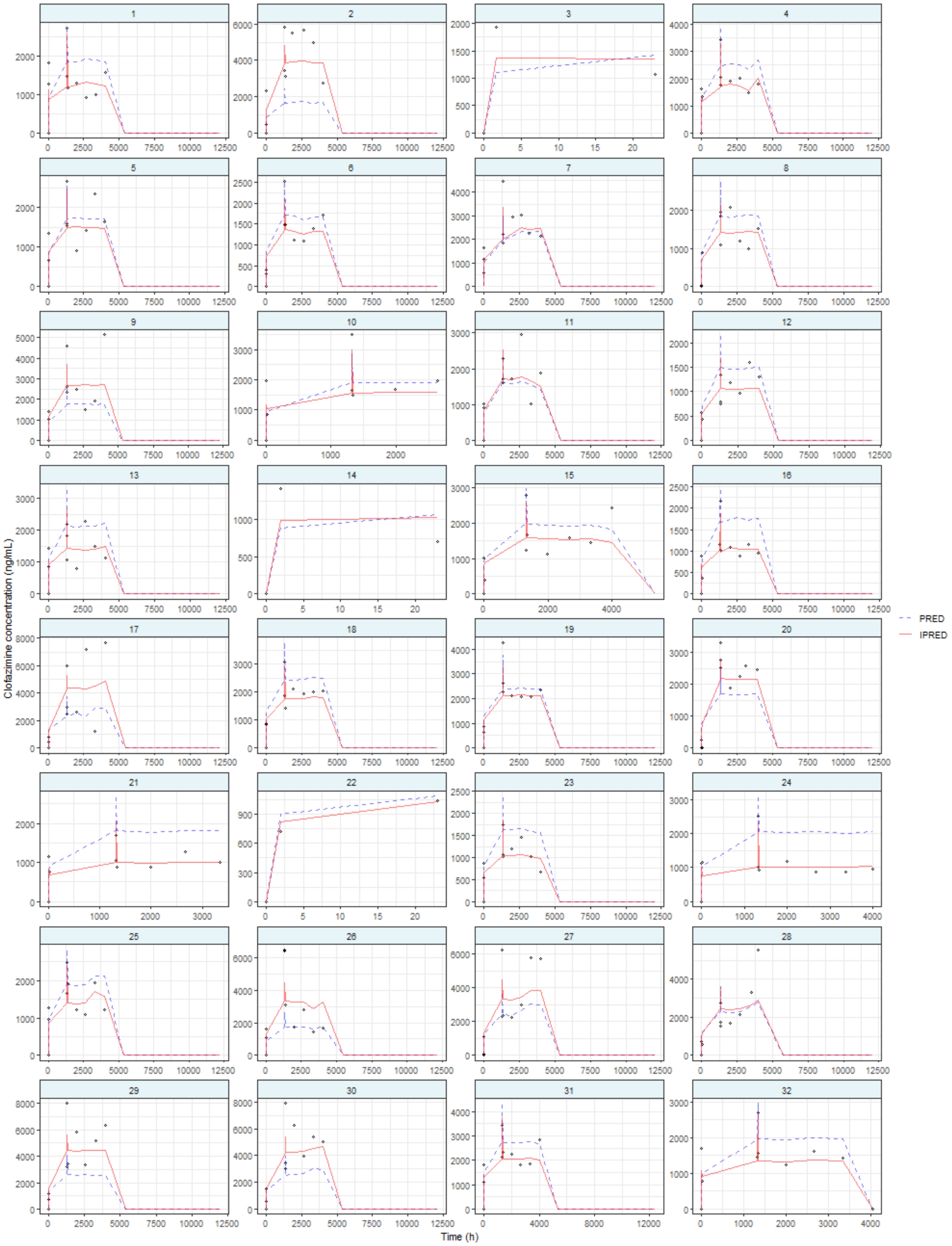

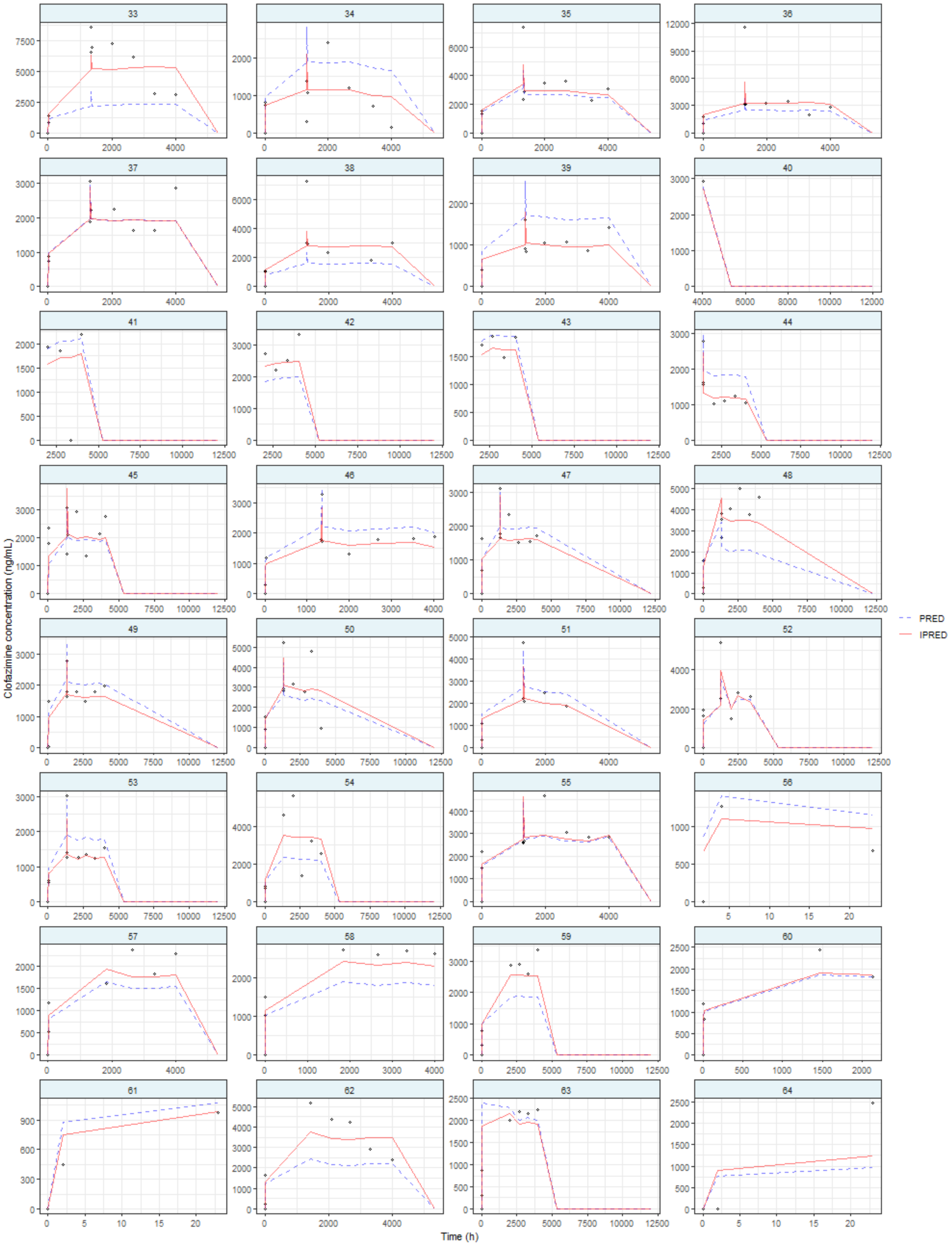
33
34
35
36
37
38
39
40
41
42
43
44
45
46
47
48
49
50
51
52
53
54
55
56
57
58
59
60
61
62
63
64
Clofazimine concentration (ng/mL)
Time (h)
PRED
IPRED

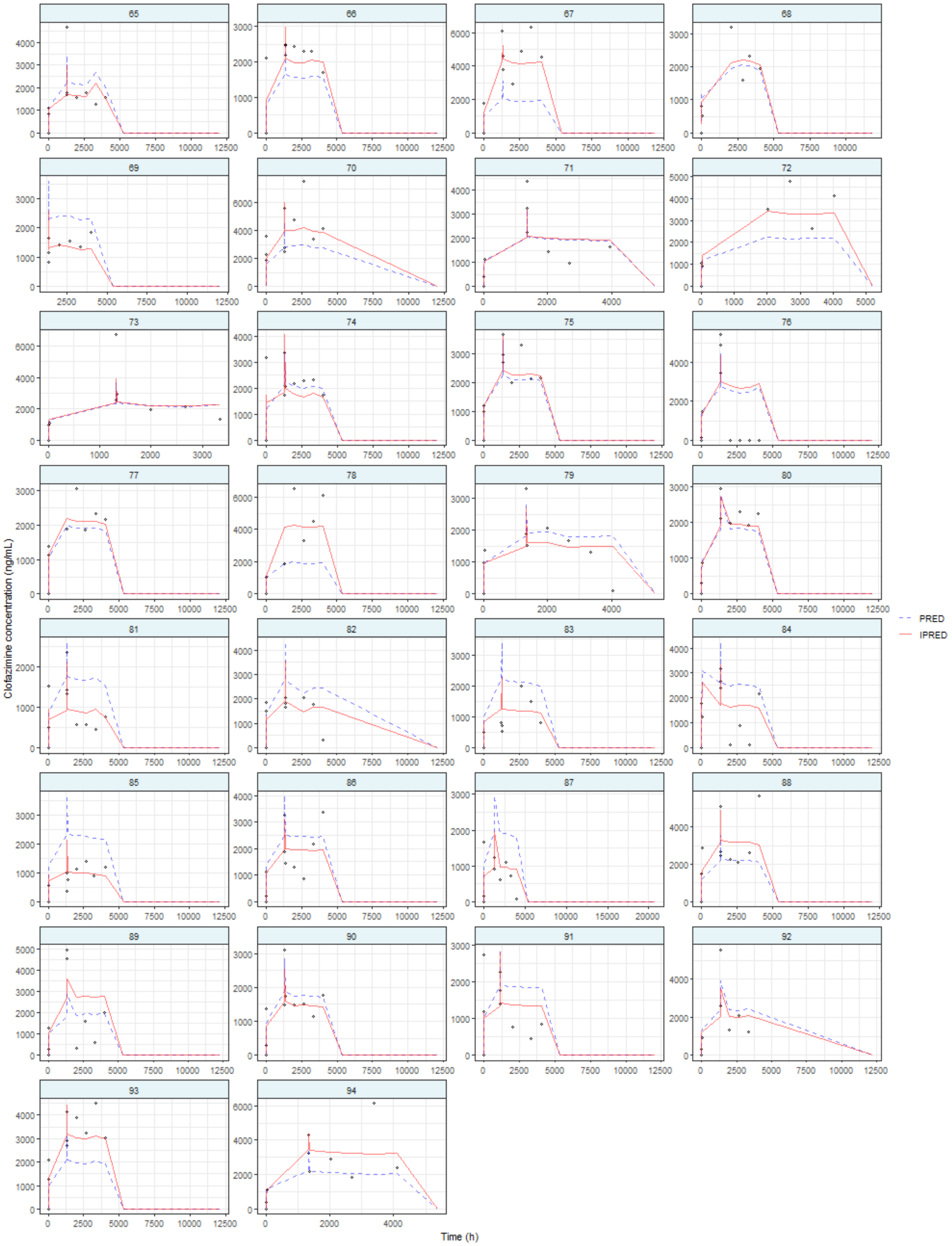

Individual linezolid plasma concentration - time profiles. Each panel represent a patient, with open circles representing observed plasma concentrations, the blue dashed line the population predictions by the developed model and the red solid lines the individual population predictions.

Appendix 4: PTA plots for a 200 mg pretomanid dose at escalating MIC's using varying plasma protein binding scenarios

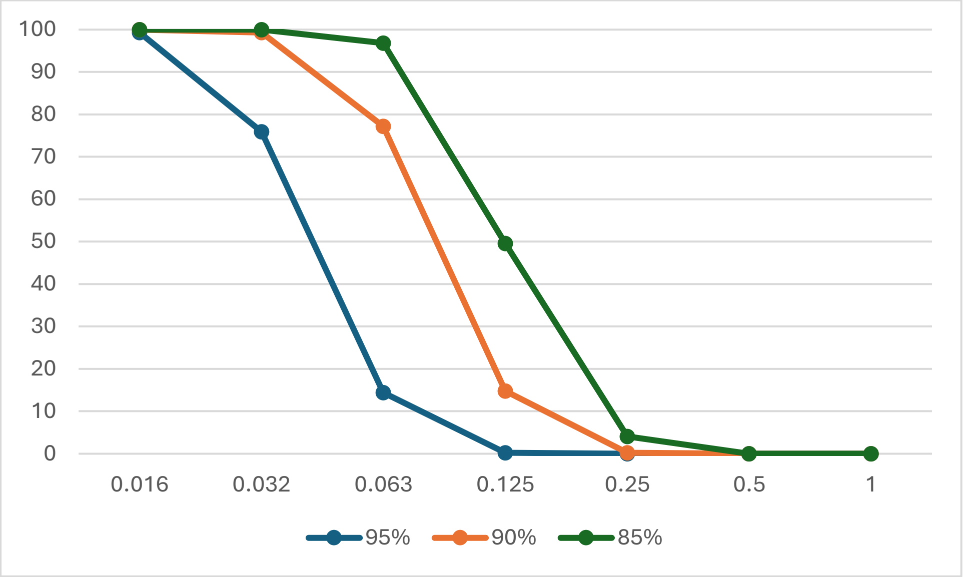

Probability of attaining an $fAUC_{0\text{-}24}$/MIC of 167 for a 200mg dose, varying protein binding assumptions.

Appendix 5: Used r packages

library(rxode2)

library(nlmixr2)

library(reshape2)

library(ggplot2)

library(tidyverse)

library(PerformanceAnalytics)

library(psych)

library(dplyr)

library(GGally)
